# Effect of Heat-Treated Garlic (*Allium sativum* L.) on Growth Parameters, Plasma Lipid Profile and Histological Changes in the Ileum of Atherogenic Rats

**DOI:** 10.3390/nu14020336

**Published:** 2022-01-13

**Authors:** Katarzyna Najman, Anna Sadowska, Krzysztof Buczak, Hanna Leontowicz, Maria Leontowicz

**Affiliations:** 1 Department of Functional and Organic Food, Institute of Human Nutrition Sciences, Warsaw University of Life Sciences, Nowoursynowska 159c, 02-776 Warsaw, Poland; anna_sadowska@sggw.edu.pl; 2 Department of Surgery, Faculty of Veterinary Medicine, Wroclaw University of Environmental and Life Science, Pl. Grunwadzki 51, 50-366 Wroclaw, Poland; krzysztof.buczak@upwr.edu.pl; 3 Department of Physiological Sciences, Institute of Veterinary Medicine, Warsaw University of Life Sciences, Nowoursynowska 159, 02-776 Warsaw, Poland; hanna_leontowicz@sggw.edu.pl (H.L.); maria_leontowicz@sggw.edu.pl (M.L.)

**Keywords:** garlic (*Allium sativum* L.), thermal treatment, Wistar rat, atherogenic rat, plasma lipid profile, ileum, intestinal villi

## Abstract

Dietary supplementation with raw garlic has a preventive and healing effect in cardiovascular diseases, but it could also damage the intestinal mucosa, resulting in impairment of nutrient absorption. Garlic processing, including heat treatment, changes the content and biological activity of garlic, so it is crucial to find food-processing methods that will preserve the health-promoting properties of garlic while minimizing its negative impact on the digestive system. Therefore, in this study, the effect of garlic (*Allium sativum* L.) on growth parameters, plasma lipid profile, and morphological parameters in the ileum of Wistar rats subjected to various types of heat treatment (90 s blanching garlic, 10 min boiling in water, 10 min pan frying without fat, microwave heating fresh garlic, 90 s blanching and microwave heating garlic, 10 min boiling in water and microwave heating garlic, and 10 min pan frying without fat and microwave heating garlic) was determined in an atherogenic diet (containing 1% addition of cholesterol). In the conducted research, it was found that the diet supplemented with heat-treated garlic used in the atherogenic diet improved the consumption and growth parameters of rats, depending on the type and time of its use. The highest consumption was recorded in atherogenic groups supplemented with garlic subjected to a longer (10 min) heat treatment and was then heated in a microwave oven. Garlic subjected to the shortest heat treatment proved to be most effective, and a significant improvement in the lipid profiles of rats’ plasma with atherogenic was observed. Extending the time of heat treatment of garlic and, additionally, its microwaving significantly weakened the action of garlic in the body, but still retained its hypolipidemic effect. The greatest influence on the structural changes in the mucosa of the rats’ iliac intestine, manifested by degeneration of the mucosa, shortening the length of the intestinal villi, damage to the brush border, and thus impairment of the intestinal absorption, was exerted by supplementing the atherogenic diet with garlic subjected to short-term heat treatment. Among the processes used, blanching was the least favorable, and the long-lasting thermal processes (cooking, frying for 10 min) had a positive effect on the mucosa of the rats’ intestines. The results obtained in this study confirm that the selection of an appropriate method of thermal processing of garlic may allow for the maintenance of preventive and therapeutic efficacy of garlic in cardiovascular diseases, while ensuring the safety of its long-term use in the context of degenerative changes in the gastrointestinal tract.

## 1. Introduction

From the dawn of history, garlic (*Allium sativum* L.) played a special role in folk medicine as a means of treating infectious diseases, fighting parasites, bacteria, fungi, viruses and the infections of the respiratory tract, skin, and food ailments caused by them [1,2,3]. Garlic provides many medicinal substances, such as flavonoid compounds, amino acids, saponins, sugars, mucus compounds, vitamins, numerous mineral salts, and microelements [4,5]. Medicinal properties of garlic, incl. antitumor [6], antidiabetic [7], anti-inflammatory and immunomodulatory [8], antimicrobial [9], and cardioprotective [10] are attributed to essential oils containing organosulfur compounds, responsible for both the unique taste and aroma of this plant, but above all for its antioxidant properties, decisive for pro-health values [11].

A well-known preventive and therapeutic effect of garlic is its effect on the circulatory system [12,13]. Garlic, by lowering the content of triacylglycerols, total cholesterol and its LDL fraction, improves the lipid profile of the blood plasma [14,15]. It also lowers blood pressure [16], and works as an anticoagulant, anti-aggregation, and fibrinolytic agent [13]. Thanks to its antioxidant properties, it prevents the oxidative modification of the LDL cholesterol fraction, and by demonstrating the ability to scavenge free radicals, interrupt free radical reactions, or enhance the activity of the body’s natural antioxidant enzymes, it protects against oxidative stress and the resulting civilization diseases, such as heart attacks, congestion, arterial hypertension, ischemic strokes, coronary artery disease, or atherosclerosis [12,17,18,19,20,21]. These properties have been demonstrated in numerous in vitro and in vivo studies, confirming the effectiveness of garlic in reducing the risk factors of many civilization diseases in clinical trials [22].

According to the scientific literature, excessive and prolonged consumption in particular of fresh garlic [23] may increase the activity of liver enzymes and exert hepatotoxic effects [24], or have a cytotoxic effect on other cells and tissues, including lungs, heart, stomach, and intestines [25], leading to severe dysfunction and damage to these organs, and even death of animals [26]. Garlic may increase the activity of some tissue enzymes, including acid phosphatase (ACP), alkaline phosphatase (ALP), and lactate dehydrogenase (LDH) in the small intestine and in the blood serum of rats [27], and thus have an adverse effect on the functioning of the intestines, causing many disorders of the gastrointestinal tract. It may also lead to the development of inflammation, ulceration of the gastric and intestinal mucosa, impairment of the absorption processes in the small intestine (mainly due to damage), atrophy [28], and shortening the length of the villi [29].

The results of studies on the effects of garlic in the prevention or treatment of gastrointestinal ailments available in the literature are insufficient, incomplete, and sometimes even contradictory. In addition, they focus on garlic used in various forms (fresh, raw, extract, extract, oil, lyophilizate, or supplements containing garlic bioactive compounds such as allicin) in various doses and at different times, or on different research models [27,30,31,32,33].

The recently published results of our research on the effect of freeze-dried raw *Alliaceae* vegetables on the morphological parameters of the hip intestine of rats fed with an atherogenic diet showed that garlic supplementation had the best anti-atherogenic effects compared to white and red onions (*Allium cepa* L.), but it significantly damaged intestinal mucosa, which in turn was accompanied by deterioration of growth parameters, resulting from the impairment of nutrient absorption in the intestine [29]. This study confirmed the negative impact of garlic on the structure of the mucosa of the ileum of rats, manifested by degeneration of the sheath mucosa, erosion of the intestinal villi, damage to the brush border, and thus impairment of the intestinal absorption function [29]. In the available literature, no other studies on the problem discussing the above-mentioned study were found.

Taking into account the obtained results, the planned research on the effect of heat treatment of garlic on the digestive system in rats of the same breed seems advisable, while taking into account that heat treatment may change the content of thermolabile bioactive components in garlic. Therefore, it is extremely important to find such thermal processes that will allow the physiologically beneficial effects of garlic consumption in the body to be maintained while limiting the occurrence of adverse changes in the digestive tract. No information was found in the available literature on the effect of garlic (*Allium sativum* L.) subjected to various heat treatment processes on the morphological changes of the intestine. Therefore, in this study, the impact of thermal processing (blanching, cooking, frying, microwave) on the content of selected bioactive ingredients and the antioxidant potential of garlic was determined, as well as the impact of the addition of freeze-dried raw and thermally treated garlic in an atherogenic diet (containing 1% cholesterol addition) on growth parameters, atherosclerotic indices, the development of plasma antioxidant potential, and morphological parameters in the iliac intestine of Wistar rats.

## 2. Materials and Methods

### 2.1. Plant Research Material

Fresh garlic (*Allim sativum* L.) of the ‘Harnaś’ variety was harvested from an arable field in the Wielkopolskie province (Kokanin, Żelazków, Poland). The bulbs were stripped of inedible parts, washed, crushed into small pieces of similar size, divided into 8 groups, and weighed.

#### Garlic and Its Treatment

Fresh garlic samples were subjected to various heat treatment processes (the most frequently used culinary techniques in traditional cuisine), which are presented in Table 1, which also includes the abbreviations used to describe them throughout the study.

Cooking and blanching of fresh garlic was carried out in boiling (100 °C) water for 10 min and 90 s, respectively (from the moment of boiling). Then, it was drained on a sieve, cooled, and weighed. The garlic was fried in a hot, fat-free pan for 10 min, and was then weighed after it cooled down. The material obtained in this way was divided into 2 parts, 1 of which (for each of the heat treatment processes used) was closed in plastic hermetic containers (intended for microwave processing) and frozen (−20 °C). Microwaving was performed in a microwave oven (Whirlpool JET 900 W, VIP 27, Whirlpool Poland Ltd., Warsaw, Poland). The frozen material was first thawed for 18 min at the power of 160 W, and then heated to a temperature of 65–70 °C (corresponding to the temperature of served meals) for 6 min at the power of 500 W. The temperature of the microwaved material was measured immediately after heating, and the microwaving process parameters were measured and established after 5 initial trials to standardize the process. After microwaving, the material was cooled again, and then weighed and frozen for further testing.

All garlic samples were frozen and then lyophilized (CHRIST ALPHA 2-4 LDplus Freeze Dryer, Martin Christ Gefriertrocknungsanlagen GmbH, Osterode am Harz, Germany). The freeze-drying process was carried out for 72 h (the pressure in the chamber was 10 Pa, the drying temperature was −50 °C, and the temperature of the shelves was 21 °C). After drying, the garlic was weighed and was brought to a powder consistency using a laboratory grinder (Grindomix GM 200, Retsch GmbH, Haan, Germany). The material was then packed into Falcone tubes and at −20 °C until testing.

### 2.2. Bioactive Compounds in Fresh and Thermally Processed Garlic (Allium sativum L.)

#### 2.2.1. Garlic Extraction

Freeze-dried garlic (100 mg) was defatted and deproteinized with acetonitrile (Pol-Aura Chemical Reagents, Zabrze, Poland), and then washed with dichloromethane (Pol-Aura Chemical Reagents, Zabrze, Poland). The samples were weighed into 50 mg screw cap tubes, and 5 mL of 1.2 M HCl in 50% methanol (Pol-Aura Chemical Reagents, Zabrze, Poland) was added. Samples with the extraction mixture were vortexed (Wizard Advanced IR Vortex Mixer, VELP Scientifica Srl, Usmate, Italy) for 1 min, incubated (IKA KS 4000 Control, IKA^®^ Poland Ltd., Warsaw, Poland) for 3 h (90 °C, shaking every 30 min), and then cooled. Samples were diluted with methanol to a volume of 10 mL and then centrifuged (MPW-380R, MPW Med. Instruments, Poland, Warsaw) (10 min, 5 °C, 6000 rpm). A clear supernatant was used to determine the antioxidant activity and bioactive ingredients.

#### 2.2.2. Total Polyphenols

The methods of Singleton and Rosii (1965) [33] were used to determine the total content of polyphenols. The absorbance of the prepared samples was measured with a spectrophotometer (UV-VIS UV-6100A, Metash Instruments Co., Ltd., Shanghai, China) at a wavelength of λ = 765 nm. The results were expressed as mg of gallic acid (Sigma-Aldrich, Poznań, Poland) equivalent per 1 g of dry matter (mg GAE/g d.m.) of garlic.

#### 2.2.3. Flavonoids

The content of flavonoids was determined in accordance with Singleton et al., (1999) [34] method. After flavonoids were extracted with 5% NaNO_2_, 10% AlCl_3_ · 6H_2_O, and 1 M NaOH (Pol-Aura Chemical Reagents, Zabrze, Poland), the absorbance of the solutions was measured with a spectrophotometer (UV-VIS UV-6100A, Metash Instruments Co., Ltd., Shanghai, China) at a wavelength of λ = 510 nm with catechin (Sigma-Aldrich, Poznań, Poland) as a standard. The results were expressed as mg of catechin equivalent per 1 g of dry matter (mg CE/g d.m.).

#### 2.2.4. Flavanols

The content of flavanols was determined according to the method of Feucht and Polster (2001) [35] using the DMACA reagent (cinnamic p-dimethylaminoaldehyde) (Sigma-Aldrich, Poznań, Poland). The absorbance was measured with a spectrophotometer (UV-VIS UV-6100A, Metash Instruments Co., Ltd., Shanghai, China) at a wavelength of λ = 640 nm with catechin (Sigma-Aldrich, Poznań, Poland) as a standard. The results were expressed as µg of catechin equivalent per 1 g of dry matter (µg CE/g d.m.) of garlic.

#### 2.2.5. Antioxidant Activity

The antioxidant activity was measured according to the Ozgen et al., (2006) [36] method using ABTS^•+^ cation radicals (2,2′-azino-bis- (3-ethyl-benzothiazoline-6-sulfonic acid diammonium) salt) (Sigma-Aldrich, Poznań, Poland). ABTS^•+^ radical solution was prepared 24 h before making the determinations by mixing inactive ABTS radicals (7 mM/L) with K_2_S_2_O_8_ (2.45 mM/L) salt (Sigma-Aldrich, Poznań, Poland). After the solution preparation, it was stored for 24 h in the dart at room temperature. Immediately before making the determinations, the ABTS^•+^ solution was diluted with methanol until an absorbance of 0.7 was obtained. The specific samples were prepared by adding 990 µL of the ABTS^•+^ solution with an absorbance equal to 0.7 to 10 µL of the extract (0.2 mg/L). After a 6 min incubation, the absorbance of the samples was measured (UV-VIS UV-6100A spectrophotometer, Metash Instruments Co., Ltd., Shanghai, PR China) at a wavelength of λ = 734 nm with Trolox (Sigma-Aldrich, Poznań, Poland) as standard. The results were expressed as µmol of Trolox equivalent per 1 g of dry matter (µmol TE/g d.m.) of garlic.

### 2.3. Animal Study

The study was conducted after approval for animal testing was granted by the Animal Care Committee of the Warsaw Agricultural University, Warsaw, Poland. Male Wistar rats from Breeding Laboratory Animals in Brwinów (veterinary identification number 14216203) were purchased for the study.

#### 2.3.1. Experimental Design

The research involved 60 rats with an average initial body weight of 127.43 ± 11.93 g, approx. 8 weeks old. The period of adaptation of animals to the diet lasted for 5 days. During this time, all rats were fed with a semi-purified control diet (C), consisting of minerals (AIN-93-MX No. catalog of mineral mix 960400), vitamins (AIN-93-VX Vitamin Blend Catalog No. 960402), mixtures, cellulose, casein, wheat starch, and soybean oil. The animals were housed in metabolic cages (TECNIPLAST S.p.A., Buguggiate, Italy) in the laboratory air-conditioned room (temperature 24 °C ± 0.5 °C, humidity 50%) over the daily light cycle of 12 h/12 h and with the illuminance of 118 lux. During the experiment (28 days), the animals in the control group (C) were fed with the control diet (C) only, and the atherogenic control group (CH) was fed with the control diet with 1% analytical grade non-oxidized cholesterol (CH) (Sigma Chemical Co., St Louis, MO, USA). The rest of the animals were fed with CH-diet supplemented with lyophilized fresh (CHGF) or thermally processed garlic in amounts depending on the weight gain of the animals during the experiment, ranging from 25 to 45 mg/day, corresponding to approx. 500 mg of fresh garlic per 1 kg of body weight of rats. Appropriate amounts of cholesterol were mixed with C diet (1:99) prior to feeding. Feeding was performed once a day at 10.00 am ad libitum. All rats had unlimited access to drinking water. Feed intake was monitored weekly by their daily and body weight. The arrangement of the experimental groups and the abbreviations used to describe the individual groups of animals are presented in Table 2.

#### 2.3.2. Animal Sacrifice

The animals were sacrificed 24 h after the last feeding with Halothan (Narcotan, Zentiva Poland Ltd., Warsaw, Poland). Blood samples were taken from the left atrium of the heart, and plasma and serum were prepared for further studies. Organs were collected, including the liver for the evaluation of the liver somatic index (SI-L), and samples of the rat ileum were used to evaluate its morphometric parameters in ileum.

### 2.4. Sampling and Measurements

#### 2.4.1. Basic Experimental Data

The body weights (BW) of the rats were measured before the start of the experiment, then once a week, and at the end of the study. The feed intake (FI) was monitored each day. The feed efficiency ratio (FER) was calculated as a gram of feed intake per gram of body weight gain (g/g). Somatic index of liver (SI-L), expressed as a percentage of body weight (% of BW), was calculated as the ratio of liver weight to final body weight of rats.

#### 2.4.2. Plasma Lipid Profile

The total cholesterol (TC), low-density lipoprotein (LDL-C), high-density lipoprotein (HDL-C), as well as triglycerides (TG) content was measured by a Siemens analyzer (ADVIA^®^ 1650 Chemistry System by Bayer^®^ Clinical Methods, Labexchange—Die Laborgerätebörse GmbH, Burladingen, Germany).

#### 2.4.3. Preparation of the Ileum Tissue Samples for Morphometric Analysis in Light Microscopy

The ileum samples for the histological evaluation of morphometric parameters were prepared as previously described by Najman et al., (2021) [29]. The collected intestinal fragments were rinsed with saline (0.9% NaCl, Pol-Aura Chemical Reagents, Zabrze, Poland) and fixed in 10% buffered formalin (pH = 7.2) (Chempur^®^, Piekary Śląskie, Poland). In a graded series of ethanol (Pol-Aura Chemical Reagents, Zabrze, Poland), they were then infiltrated with xylene (Pol-Aura Chemical Reagents, Zabrze, Poland) and in liquid paraffin (Pol-Aura Chemical Reagents, Zabrze, Poland) and carousel tissue processor (STP 120 Spin Tissue Processor, Especialidades Médicas MYR, S.L., Lleida, Spain). Finally, they were embedded in paraffin wax using modular tissue embedding center (MICROM EC350-2, MICROM, Thermo Fisher Scientific Inc., Waltham, MA, USA). After that, tissue samples were cut at 5 µm thick sections with the use of rotary microtome (MICROM Section-Transfer-System HM325, MICROM, Thermo Fisher Scientific Inc., Waltham, MA, USA), placed on glass slides, and dried in laboratory thermostat (WAMED SSP, Warsaw, Poland) at 40 °C for 12 h. Routine staining of slides with hematoxylin-eosin (HE) (Pol-Aura Chemical Reagents, Zabrze, Poland) (Pol-Aura Chemical Reagents, Zabrze, Poland) was made and then the slides were closed in Roti^®^-Histokitt (Carl Roth, Linegal Chemicals Ltd., Warsaw, Poland).

#### 2.4.4. Light Microscope Assessment

Images of intestinal tissue fragments were assessed with a light microscope (OLYMPUS BX61, Olympus Poland Ltd., Warsaw, Poland) equipped with a camera, computer software (Cell^P imaging software, Olympus, Hamburg, Germany), and a stage micrometer. In each slide, the height of six clearly visible villi and depth of six intestinal crypt, as well as the thickness of the mucosa and muscles, were measured, and were randomly selected from six different areas of each tissue harvested. From the obtained results, mean values were calculated for each rat and the experimental group.

### 2.5. Statistical Analysis

The results obtained in the study are presented as mean values ± SD (standard deviation) from six measurements (*n* = 6) and analyzed by one-way ANOVA using Tukey’s multiple range test (the assumption of equal variance) or the Gomes–Howell multiple range test (no assumption of equal variance). The SPSS Statistics version: 28.0.1.0 (142) (IBM, Armonk, NY, USA) statistical program was used to perform a statistical analysis of the research results obtained in the work. Differences were considered to be significant at *p* < 0.05.

## 3. Results and Discussion

### 3.1. Bioactive Compounds in Fresh and Thermally Processed Garlic (Allium sativum L.)

The content of selected bioactive compounds (total polyphenols, flavonoids, flavanols) and the antioxidant activity of freeze-dried fresh and thermally processed garlic are presented in Table 3. It should be noted that, in the current work, for comparative purposes, only recently published results for fresh, non-heat-treated garlic (GF) were used, because the results presented in this paper are a continuation of partially published studies [29].

The highest content of total polyphenols, flavonoids, and flavanols was found in fresh (GF) and microwave (GF micro) garlic. Under the influence of the applied heat treatment, the content of phenolic compounds decreased, depending on the type of culinary treatment used. No differences were noticed between the total polyphenols content in garlic subjected to blanching, frying, blanching, and microwaving, as well as frying and microwaving. Losses of polyphenols content in these samples oscillated around 17–22%. Short-term heat treatment, i.e., microwaving fresh garlic (GF micro), as well as blanching (G90s) and frying in a pan for 10 min (G10P), did not cause changes in the content of flavonoids. These treatments influenced the content of flavanols. Frying generated losses of these compounds at the level of approx. 40% (G10P), blanching 23% (G90s), and microwave alone contributed to approx. 12% (GF micro) of losses. The greatest losses in the content of these components were found in garlic cooked for 10 min (G10) and in samples cooked and microwaved (G10 micro). Losses in the case of the total content of polyphenols ranged from approx. 30 to 38%, and, in the case of flavonoids and flavanols, respectively, from approx. 45 to 48% and from approx. 40 to 54%.

Literature data on the content of polyphenolic compounds in *Alliaceae* plants are characterized by great variability, due to the variety of extraction mixtures, as well as the extraction methods used. The evaluation of the obtained results of the content of polyphenolic compounds is correlated with the extraction procedures used [37]. Nevertheless, the results of the content of phenolic compounds in garlic obtained in this study are comparable with the data in the literature, in which the total polyphenol content for garlic was on average 18.9 mg GAE/g d.m. [4,5,38,39].

In the conducted research, a decrease in the content of total polyphenols, flavonoids, and flavanols was found during the extended time of thermal treatment. The observed trends are confirmed by studies by other authors who suggest that technological treatments may change the phenolic compounds contained in garlic or other plants from the *Alliaceae* family [40,41]. Blanching for 90 s (G90s) and microwaving raw (GF micro) or blanched garlic (G90s micro) used in the present study led to the lowest loss of bioactive compounds, and the results obtained for heat-treated garlic were comparable with the results of other authors [42]. The greatest changes occurred during the thermal processing lasting 10 min, i.e., pan frying (G10P) and boiling in water (G10), which is also confirmed by the studies of other authors [38,39].

In this study, the decrease in the content of phenolic components during the applied heat treatments was accompanied by a decrease in the antioxidant potential of garlic (Table 3). The highest antioxidant activity was observed in fresh garlic GF and garlic subjected only to microwaving (GF micro), while both blanching (G90s) and pan-frying (G10P), as well as microwaving blanched and fried garlic (G90s micro and G10P micro), lowered its antioxidant properties (on average to 37.62 ± 1.81 µmol TE/1 g d.m.) by about 21% compared to the fresh GF garlic. Undoubtably, the lowest antioxidant activity was observed, similarly to the content of polyphenols, in garlic boiled for 10 min in water (G10) and additionally microwaved, for which the values of this parameter were 36% and 43% lower than in the GF, respectively.

According to many authors, during processing, mainly thermal, compounds contained in plants may undergo significant changes, often leading to a reduction in their bioavailability or biological activity [38,39,40], which may also be accompanied by a reduction in the antioxidant activity of plants, and thus a reduction in their pro-health effects in the body. Some studies suggest that heating destroys the structure of active sulfur compounds in garlic and causes a significant decrease in the content of polyphenols in *Alliaceae* plants [39,41,42].

Therefore, it is of great practical importance to assess the impact of thermal processing used during food preparation on changes in the content of biologically active compounds and the antioxidant potential of onion vegetables consumed on a daily basis. This is because it helps to indicate the types of culinary treatments that maximally protect compounds with beneficial effects on health contained in fresh vegetables, thus ensuring the maximum therapeutic effect of these plants.

### 3.2. Animal Study

#### 3.2.1. Feed Intake and Diet Performance Indices in Rats Supplemented with Freeze-Dried Fresh and Thermally Processed Garlic

In this study, the influence of fresh garlic subjected to various heat treatment processes (blanching, cooking, frying, microwave cooking) on the physiological parameters in rats fed with a semi-synthetic and/or atherogenic diet (with 1% cholesterol) and supplemented with freeze-dried garlic was assessed. The results of diet, cholesterol, and freeze-dried garlic consumption, as well as the results of growth parameters, including body weight gain, feed efficiency ratio (FER), and the somatic index of the liver (SI-L) after 28 days of the experiment, are presented in Table 4. It should be mentioned that, in the current work, for comparative purposes, the recently published results for the control groups (C and CH) and for fresh, unprocessed garlic (CHGF) were used; therefore, the results presented in this paper are a continuation of the already partially published research [29].

As shown in Table 4, the mean dietary consumption of all experimental animals was 584 ± 81 g. Based on the data obtained (Table 4), it can be concluded that the animals fed with a diet with 1% addition of cholesterol (CH), supplemented with freeze-dried fresh garlic (CHGF), and subjected to “single” heat treatment (CHG90s, CHG10P, CHG10) showed significantly lower feed intake, and that the addition of cholesterol and freeze-dried garlic to the diet of animals supplemented with garlic subjected to an additional microwave process (“micro” groups) was most likely due to the very intense aroma of food and reluctant consumption by animals. The use of additional microwaving in all previously thermally treated garlic samples, in all groups receiving “micro” garlic, improved feed consumption, and thus also the addition of cholesterol and garlic freeze-dried improved food consumption on average by 38%, 38%, and 29%, compared to the CHGF group, respectively. The lower consumption of a diet supplemented with fresh garlic could be due to the strong and irritating aroma of garlic.

According to the literature, the processes of heat treatment of garlic can change the profile of organosulfur compounds. The allicin, which is responsible for the sharp, typical taste of raw garlic and is unstable at high temperatures, is transformed to other, less irritating organosulfur compounds during longer heat treatment, which in turn reduces the content of volatile compounds [42]. This may explain the variation in the consumption of a diet supplemented with freeze-dried garlic additives in this experiment.

Varying dietary intake resulted in differential body weight gain in the animals during the 28 day experiment. As seen in Table 4, the addition of freeze-dried fresh thermally processed garlic used in the atherogenic diet (CH) influenced the body weight gain of animals in a manner dependent on the type and duration of heat treatment of garlic. The lowest BWG was shown by animals receiving the addition of fresh garlic (CHGF) and garlic subjected to “single” heat treatment (CHG90s, CHG10, CHG10P), where it was, on average, 13.9 ± 23.5 g, while the highest came from animals fed with a CH diet supplemented with microwaved garlic (mean 201.3 ± 21.2 g), with the best BWG in the CHG10P micro group.

Despite the varied consumption, no differences were found in the somatic index of the liver (SI-L) and in the feed efficiency ratio (FER) (Table 4). More favourable results in the feed efficiency ratio were found in rats fed CH diet with the addition of freeze-drying, subjected to “double” thermal treatment of garlic, for which FER averaged 3.33 ± 0.32 g/g, and was lower than in the animals supplemented with freeze-dried “single” heat-treated garlic, i.e., CHG90s, CHG10, and CHG10P (reaching mean FER values of 3.90 ± 0.38 g/g).

There are few similar studies in the available literature, and they usually relate to a longer period of feeding animals on a high-fat diet. The studies of other authors confirm the tendency in our studies to reduce the fat content in the liver of animals as a result of supplementation with garlic powder [43] or garlic oil [44]. Gorinstein et al., (2011) [21] showed a significant reduction in the optical density of fat in rat hepatocytes, as well as a significant reduction in the formation of atherosclerotic lesions in the aorta of rats fed with an atherogenic diet enriched with raw onion vegetables, including garlic, in an experiment lasting 6 weeks. However, the authors did not show either an increased degree of fatty liver or atherogenic changes in the histological picture of the aorta, heart, or brain of animals in a shorter (4-week) experiment, which can be explained by an excessively short period of loading rats with cholesterol [20]. Presumably, only the longer influence of the atherogenic diet, resulting in increased lipogenesis in the organism and a large excess of lipids in the bloodstream, changes the direction of their transformations, not only causing their accumulation in the bloodstream but also in other organs, e.g., in the liver or aorta [21].

#### 3.2.2. Lipid Profile in Rats Supplemented with Freeze-Dried Fresh and Thermally Processed Garlic

**Figure 1:**
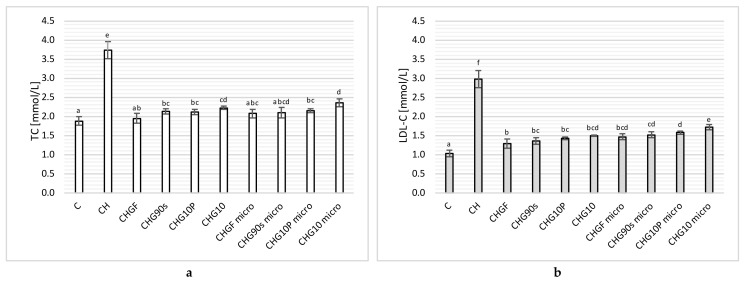
Influence of freeze-dried fresh and thermally processed garlic on plasma lipid profile: total cholesterol (TC—**a**) and LDL cholesterol (LDL-C—**b**) content in rats fed control- and cholesterol-containing (1% of cholesterol) diet during the 28 day experiment. Values are means ± SD (*n* = 6). Means in bars with different letters are significantly differ (*p* < 0.05); Tukey’s test.

In animal studies, the effect of dietary nutrition with or without cholesterol and an addition to the atherogenic (CH) diet of fresh freeze-dried and subjected to various heat treatment processes of garlic on the lipid profile in rats was investigated, and the obtained results are shown in Figure 1.

The administration of an atherogenic diet (CH) to rats worsened the plasma lipid profile (Figure 1), causing an increase in total cholesterol (Figure 1a) and its LDL-C fraction (Figure 1b) in the blood serum of rats compared to the control diet (C) without its participation by almost two (TC) and three times (LDL- C). The addition of freeze-dried fresh and heat-treated garlic used in the CH diet improved the lipid profile in a manner dependent both on the type of process used and its duration.

Fresh garlic showed the best hypolipidemic effect, both in relation to TC and LDL-C, and lowered the content of these lipids by approximately 48% and 57%, respectively, compared to the CH group. Thermal treatment weakened the effect of garlic on the lipid profile, while blanching (CHG90s) and pan-frying (CHG10P) retained its hypolipidemic properties to the greatest extent, significantly reducing the content of TC and its LDL-C fraction in the blood by 43% and 53%, respectively, compared to CH. Fresh garlic subjected to microwave only (CHGF micro) showed similar properties, reducing serum lipid levels by about 45% and 51% for TC and LDL-C, respectively. The least effective hypolipidemic effect was shown by garlic boiled in water for 10 min (CHG10), which lowered the level of TC and LDL-C by 41% and 50%, respectively, but nevertheless retained its beneficial properties in regulating the body’s lipid metabolism. The TC content in the groups receiving microwaved, previously heat-treated garlic (CHG90s micro and CHG10P micro) decreased by about 43% and 48%, respectively, compared to the CH group, indicating that this process did not reduce the hypolipidemic properties of garlic. The exception was in the CHG10 micro group, showing the weakest effect on the plasma lipid profile, reducing the content of TC and LDL-C by 37% and 43%, respectively.

In the conducted experiment, no statistically significant differences were found in the content of HDL-C fraction, both between groups C and CH, as well as under the influence of supplementation with freeze-dried raw or thermally treated garlic used in an atherogenic diet. The mean content of HDL-C fraction in all experimental animals was 0.68 ± 0.11 mmol/L, which was consistent with the results of studies by other authors [15,16,17,18,19,20].

The analysis of the available literature shows that garlic exerts a significant hypolipidemic effect in rats fed with a high cholesterol diet, reducing the level of total cholesterol, as well as its LDL-C fraction and triacylglycerols. It also improves the lipid profile of the blood plasma, without significantly affecting the HDL-C cholesterol fraction [14,22,45], which is consistent with the results obtained in our work. According to some studies, during the supplementation of an atherogenic diet with garlic, in addition to lowering TC, LDL-C, and TG, the level of HDL-C fraction also increased [46]; however, no such relationship was found in the rat experiment performed in this study.

The mechanism of the hypolipidemic action of garlic is explained in the literature by the inhibition of cholesterol synthesis at the stage of the reduction in hydroxymethyl glutaryl-CoA (HMG-CoA) under the influence of HMG-CoA reductase, the enzyme key for cholesterol biosynthesis in the body [47]. Garlic and its extracts inhibited HMG-CoA reductase activity and reduced cholesterol synthesis [48]. A similar ability to regulate the activity of the HMG-CoA enzyme was also shown by ajoenes, S-allylcysteine, and other organosulfur metabolites of garlic [49]. Studies by Gebhardt et al., (1994) [47] carried out on rat liver cells and human HepG2 cells aimed to analyze the influence of allicin and ajoen on various stages of cholesterol biosynthesis, and showed that ajoen in rat hepatocytes significantly reduced cholesterol synthesis (by 18%) with no effect of allicin noted. On the other hand, in human HepG2 cells, there was a marked reduction in cholesterol biosynthesis both under the influence of allicin (decrease by 14%) and ajoen (decrease by 19%). In both cases, the inhibition of cholesterol biosynthesis took place at the stage of the reaction carried out by the enzyme HMG-CoA reductase [47].

Hypolipidemic activity is also demonstrated by steroid saponins present in garlic [43] as well as water-soluble sulfur metabolites, such as SAC (S-allyl-cysteine) or oil-soluble metabolites, in addition to the aforementioned ajoenes, sulfides (DAS—diallyl sulphide), disulphides (DADS—diallyl disulphides), or diallyl trisulphides (DADTS—diallyl trisulphide), effectively inhibiting cholesterol synthesis [48,49].

The results obtained in this study correspond with the studies of other authors who confirmed the hypolipidemic effect of garlic [14,15,16,17,18,19,20,21]. Various preparations, in the form of a powder, extract, or dietary supplement, to a varying degree, depending on the time of supplementation and the dose of garlic, significantly improved the plasma lipid profile in rats [19,50], mice [44], or humans [13].

The biological activity of garlic largely depends on the chemical structure, content, and profile of bioactive compounds of garlic [2,5], which may change even during pre-treatment processes (such as peeling, cutting, crushing, or maceration), without which would be impossible to synthesize volatile compounds in garlic [11]. In turn, losses of biologically active ingredients under the influence of heating are related, among others, to with the rate of heat transfer into tissues. Heating in water causes the effect of temperature in the entire volume of the material; therefore, it leads to greater losses than heating in air or steam [38,39,40,41], as demonstrated in the present study.

A specific process is thermal processing in a microwave oven (microwave), mainly used to reheat ready-made and often frozen dishes or meals. The transfer of energy from electromagnetic waves to the heated material is effective only when it contains water. Due to the shorter time and the operation of a lower temperature, the microwave process leads to smaller losses of antioxidants compared to traditional methods of heating [51], which was also confirmed in the research carried out in this paper.

There is also evidence based on in vitro studies that the bioavailability of many bioactive compounds increases with heat treatment; however, information on the effect of, e.g., cooking on the nutritional value of vegetables is incomplete [51]. In the case of garlic, a known process that repeatedly increases both the content of bioactive ingredients and its antioxidant potential is long-term heat treatment (fermentation), leading to the production of black garlic [52]. Therefore, thermal processes do not have to and cannot be unequivocally equated with a negative effect on the health-promoting properties of plants, including garlic.

According to the literature, the health-promoting properties of garlic, in particular its antiatherosclerotic and hypolipidemic effects, require long-term supplementation in the diet, which may also be accompanied by undesirable, harmful, and even toxic side effects of excessive consumption, especially of raw garlic [23,29]. In addition to the unpleasant smell associated with the consumption of fresh garlic, the literature contains information about the allergic or irritating effects of garlic [1,53,54]. There are also reports on the effect of excessively high doses of garlic on lowering blood calcium levels and increasing the risk of osteoporosis, asthma disorders, spermatogenesis, or liver and heart damage [24,25].

Excessive consumption of garlic, especially raw, may also result in digestive disorders, such as excessive gas production and flatulence, heartburn, reflux, nausea and vomiting (especially when consumed on an empty stomach), abdominal pain, constipation, or diarrhea [1,26,27]. The available literature shows that an excessive supply of raw garlic in the diet leads to redness and inflammation, as well as ulceration and damage to the gastric and intestinal mucosa [55]. According to our recently published studies, supplementation of an atherogenic diet with garlic, white onions, and red onions has a different effect on the mucosa of the hip intestine of rats. While the addition of onions may improve the growth and development of the rat intestinal mucosa, the addition of raw garlic causes damage to the intestinal mucosa, impaired absorption function, and, consequently, deterioration of growth parameters in rats fed with an atherogenic diet [29].

#### 3.2.3. Morphometric Analysis of the Rats’ Ileum Fed Control- and Atherogenic Diet Supplemented with Freeze-Dried Fresh and Thermally Processed Garlic

**Figure 2:**
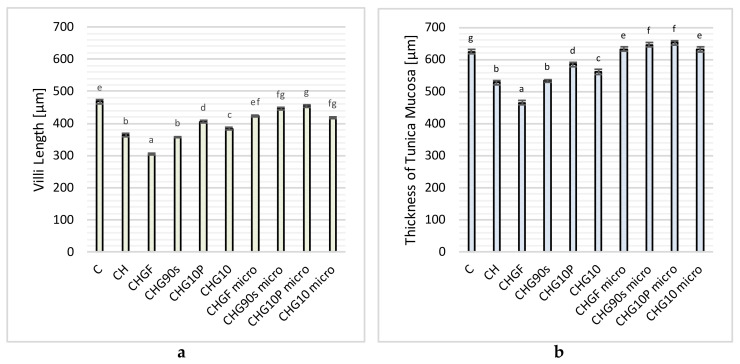
Influence of freeze-dried fresh and thermally processed garlic on villi length (**a**) and thickness of tunica mucosa (**b**) in rats fed control- and cholesterol-containing (1% of cholesterol) diet during the 28 day experiment. Values are means ± SD (*n* = 6). Means in bars with different letters are significantly different (*p* < 0.05), Tukey’s test.

Due to the fact that even slight changes in the chemical composition of bioactive compounds can cause significant differences in the bioavailability and biological activity of end products [4], it can be concluded that different methods of processing garlic preserves or contributes to the production of bioactive ingredients to a different extent, and therefore that their impact in the body will be highly diversified. Therefore, the study investigated the effect of the addition of garlic subjected to various types of heat treatment (blanching, cooking, frying, and microwaving) on morphological changes in the ileum in rats fed with an atherogenic diet. The obtained results, which are a continuation of those already published [29], are presented in Figure 2.

Following the conducted and partially published studies [29], the highest intestinal villi (468.59 ± 6.38 µm) were found in animals fed with the control diet (C), which were 29% higher than in the group of rats receiving the control diet with 1% of cholesterol (CH) (363.59 ± 4.90 µm). The use of freeze-dried fresh garlic (CHGF) in the atherogenic diet (CH) resulted in the deterioration of intestinal villi growth compared to the control groups, by approx. 16% in relation to the (CH) group and as much as approx. 35% in relation to group (C). The processes of heat treatment of garlic reduced its unfavorable effect on the growth of intestinal villi in a manner dependent on the type and time of thermal treatments used. Significantly lower (*p* < 0.05) damage to these structures was found in the case of supplementation with freeze-dried blanched garlic (357.70 ± 1.98 µm). The rats in this group (CHG90s) had approximately 17% higher villi than those supplemented with fresh garlic. Extending the heat treatment time to 10 min resulted in an improvement in this parameter, with a better growth of the intestinal villi in the group of animals supplemented with pan-fried garlic (CHG10P) (405.85 ± 3.22 μm) than those boiled in water at the same time (CHG10) (383.93 ± 4.78 µm), for which the intestinal villi reached a height of approximately 33% and 26%, respectively, higher than in the CHGF group.

Microwaving the previously thermally treated garlic improved the morphological parameters of the rats’ intestine. The average height of the intestinal villi in all groups of animals fed with the atherogenic diet with the addition of microwaved garlic (“micro” groups) was 435.90 ± 16.01 µm, and was significantly higher than in the atherogenic (CH) group (on average by about 21%) and lower by only approx. 7% than in the cholesterol-free control group (C). The highest intestinal villi in the groups supplemented with microwaved garlic were found in rats fed with the CH diet with the addition of microwaved fried garlic (CHP10P micro), whose mean value was 454.73 ± 3.67 µm.

A similar trend was observed for the thickness of the intestinal mucosa of rats. Following the previously published studies [29], the control groups differed in terms of this parameter, with a significantly thicker mucosa of the ileum found in rats fed with a control diet (C) (624.52 ± 6.98 µm) than the atherogenic control (CH), i.e., with 1% cholesterol (527.70 ± 5.95 µm). Supplementation of the atherogenic (CH) diet with freeze-dried raw garlic worsened the condition of the rat intestinal mucosa (was the lowest at 465.22 ± 7.39 µm in these rats), while the use of heat-treated garlic reduced its negative impact on these parameters.

Blanching of garlic improved the structure and increased the thickness of the intestinal mucosa (533.67 ± 3.74 µm) compared to the CHGF group, while extending the heat treatment time to 10 min resulted in an improvement in this parameter by approx. 21% (in the CHG10 group) and 26% (in the CHG10P group), suggesting that supplementation of the CH diet with fried garlic (584.40 ± 6.01 μm) had a better effect than with boiled garlic (560.81 ± 8.82 μm) at the same time.

Microwaving of garlic samples improved the growth and development of the intestinal mucosa in rats, which reached an average thickness of 640.48 ± 16.01 µm (higher than in the CH or even in C group). It should be emphasized that the most beneficial effect on the development of the intestinal mucosa of rats fed CH diet was supplementation with freeze-dried microwaved garlic which was previously fried for 10 min (CHG10P micro), for which the thickness of the intestinal mucosa was the largest (651.81 ± 7.33 µm).

**Figure 3:**
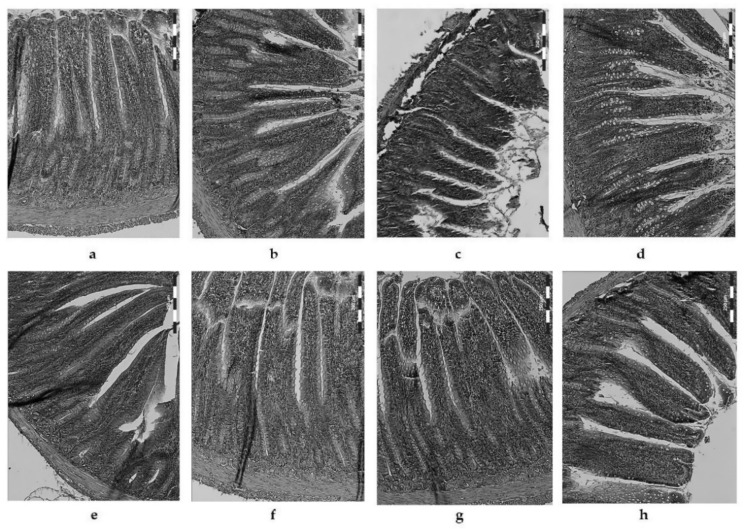
Histological examination of the rats’ ileum fed with control (C—**a**), atherogenic diet (CH—**b**) and CH diet supplemented with freeze-dried fresh (CHGF—**c**) and thermally processed garlic: 90 s blanched (CHG90s—**d**), fresh and microwaved (CHGF micro—**e**), 90 s blanched and microwaved (CHG90s micro—**f**), 10 min pan fried and microwaved (CHG10P micro—**g**), and 10 min boiled in water and microwaved garlic (CHG10 micro—**h**). Light microscope (×200).

As shown in Figure 3a, the tunica mucosa in rats receiving the control diet (C) was of a normal structure, with cells showing the appropriate integrity and structure. The microscopic examination showed that no foci of necrosis or degeneration in the intestinal villi were significantly densified, and that they were high and tightly adjacent to each other, with a finger-like shape (natural and slender), a well-marked brush border, and intact enterocytes. In atherogenic rats (CH), the shape of the intestinal villi changed (greater thickness at a lower height, with irregular ends and enterocytes protruding from the brush border line), and their degradation was noticeable (Figure 3b) [29].

The greatest changes in intestinal villi (both in their shape and height) and of the ileum tunica mucosa were observed in rats fed with a CH diet supplemented with freeze-dried raw garlic (CHGF) (Figure 3c). The villi were irregular in shape, and were much shorter, thicker, and wider. Furthermore, some were leafy, while others were lingual in shape and branched at the tips. The apices of the enterocytes were clearly flattened, dull, and damaged, with incisions present [29].

Supplementation of the atherogenic (CH) diet with freeze-dried, thermally treated garlic to a different extent improved the histological parameters of the rat hip intestine. Animals receiving the addition of freeze-dried blanched garlic (CHG90s) in the CH diet (Figure 3d) showed a slightly better structure of the intestinal villi and intestinal mucosa; however, the apical surface of the enterocytes was still flattened, damaged, and irregularly arranged in relation to the brush border line. The architecture of the intestinal villi was heterogeneous, and there were villi of a leafy and conical shape, partially flattened, and partially branched at the tops. The villus density also decreased compared to the control group (C).

Supplementation of the atherogenic (CH) diet with microwaved fresh garlic (CHGF micro) improved the height of the intestinal villi (Figure 3e), but they were still characterized by a variation in height and shape (in some cases they were blunted, and in others they tightened the apical surface of enterocytes). Moreover, as in the CHGF and CHG90s groups, a significant reduction in their density in the intestinal mucosa was observed.

Interestingly, supplementation with garlic additionally heated in a microwave oven (“micro” groups) had a much better effect on the morphology of the rat intestinal mucosa (Figure 3f–h). In the microscopic image of the intestines of CHG90s micro group (Figure 3f), the intestinal villi were closely adjacent to each other, densely arranged, dominated by structures with a typical finger-shaped and smooth surface, and their height was also improved (Figure 2).

Favorable changes were also observed in rats fed with the CH diet supplemented with garlic subjected to a longer, 10 min heat treatment (Figure 3g–h). The intestinal villi had a typical, distinctive, finger-shaped shape, and were of a similar height and thickness. They were covered with regular enterocytes with a well-developed brush border in one line. The apical part of the villi was properly developed, but in the CHG10 micro group (Figure 3h), the villi density was lower than in most of the groups supplemented with heat-treated garlic. In the CHG10P group (CH with the addition of microwaved garlic fried in a pan for 10 min), the mucosa of the rats’ intestine dominated intestinal villi of a typical structure, smooth surface, uniform shape, tightly adjacent to each other, with the same height and a uniform and dense arrangement (Figure 3g). The image of the intestinal mucosa in these animals was very similar to the image of the rats fed with the control diet (C), which clearly indicates the most beneficial effect of microwaved fried garlic on the development of the intestinal mucosa of rats fed with the CH diet.

No differences were found in the thickness of the tunica muscle or in the depth of the intestinal crypts in rats fed with the control (C) and atherogenic (CH) diets as well as under the influence of supplementation with freeze-dried raw or thermally treated garlic. The mean value for the thickness of the tunica muscle and the crypts depth in all tested animals was 63.83 ± 2.92 µm and 131.37 ± 3.10 µm, respectively.

In the available literature, no data were found on the effect of supplementation of an atherogenic diet with freeze-dried garlic subjected to various heat treatment processes on the morphological parameters of the rats’ iliac ileum. According to our recently published research [29], supplementation of the atherogenic diet with freeze-dried raw onion plants, i.e., garlic (*Allium sativum* L.) and white and red onions (*Allium cepa* L.), had a significant effect on the mucosa intestines of rats fed with a high cholesterol diet, with onion supplementation having a beneficial effect on the intestinal mucosa, improving its growth and development, as opposed to raw garlic supplementation. The addition of raw garlic led to serious damage and degeneration of the intestinal mucosa, which was accompanied by deterioration in diet utilization and growth indices in rats, most likely resulting from impairment of intestinal absorption functions [29].

A similar relationship was also noted in the current work. Along with the deteriorating morphological parameters of the intestine, resulting from various degrees of damage to the intestinal villi and degradation of the tunica mucosa, deterioration in the growth parameters of the animals was noted (Table 4). The intestinal mucosa, which plays a key role in digestion and, above all, absorption, determines the optimal use of nutrients in the body [31]. Therefore, its damage, degeneration, and disturbances within the brush border ultimately lead to impairment of both digestive functions (due to insufficient enzyme production) and absorption functions, which is manifested, among others, by limiting the growth of animals [56,57].

As in previous studies [29], excessive discharge in the form of amorphous material was observed in the lumen of rats, in particular supplemented with freeze-dried fresh, microwaved, and blanched garlic (Figure 3), which is most likely the result of an ongoing inflammatory process in the area of glandular cells and crypts and the accompanying excessive shedding of the intestinal mucosa cells. Similar results were reported by Riad et al., (2008) [31] in an experiment on mice infected with *Schistosomiasis Mansoni* and treated with a high (100 mg/kg body weight) dose of garlic.

There are reports in the available literature on the toxic effects of garlic, especially when used for a long time and in high doses, on the gastrointestinal tract of rats [27,28]. The use of high doses (5 mL/kg) of raw garlic extract in rats led to gastric damage that was severe to result in the death of the animals [26]. In studies on the toxicity of garlic, Joseph et al., (1989) [25] administered garlic extract to rats in the amount of 2 mL/100 g of body weight (intragastrically) and found serious disturbances in the secretion of liver enzymes, as well as significant congestion of this organ, while the use of garlic oil in the same amount given to animals on an empty stomach led to death. Amagase et al., (2001) [1] administered 0.5 mL of raw garlic juice to rats, which caused hyperemia, followed by ulceration, erosion, and bleeding of the intestinal mucosa. The commercial preparations with different garlic content (from 60 to 133 mg/rat) studied by the same authors also caused severe damage to the intestinal mucosa, which the authors explained by the toxicity of allicin. Kodera (1997) [55] also attributed the toxic effects to the intestinal and gastric mucosa to allicin. In other studies, a high dose of garlic powder, administered at an amount equivalent to 70 mg of fresh garlic/kg, led to degeneration, inflammation, degradation, and necrosis in the gastric wall [58].

According to the literature, the toxic effects of garlic include on the digestive tract is not only found in high doses, but also in their long-term use. Augusti (1996) [12] showed that long-term garlic supplementation was accompanied by significant anemia caused by the breakdown of erythrocytes in rats, resulting in reduced or inhibited growth and drastic weight loss. Riad et al., (2008) [31] showed the toxicity of garlic and its influence on degenerative changes in the ileal mucosa, depending on both the dose (50 mg/kg and 100 mg/kg body weight) and time of garlic treatment (administered every 2 days for 6 and 12 weeks) to treat *Schistosomiasis Mansoni* infection. The authors observed degenerative changes ranging from mild (for lower doses and shorter treatment period) to significant damage to the mucosa. Long-term therapy with high doses of garlic was accompanied by clear atrophy of the intestinal villi, degeneration, inflammation, infiltrates in the mucosa, hypertrophy of the muscular membrane and edema, as well as disturbances in the functioning of the intestinal crypts.

The results of the research on raw garlic obtained in our work are, therefore, confirmed by the studies of other authors. The available literature provides little information on the toxic effects of raw garlic on the gastrointestinal tract (especially when administered in high doses or when used long-term), and lacks information on the possibility of eliminating the harmful or toxic effects of garlic on the gastrointestinal tract, which is the key issue for safe, long-term supplementation necessary to achieve both prophylactic and therapeutic properties of this plant.

Our research, both in this and recently published papers [29], showed that the very load with a high-cholesterol diet had a negative effect on the mucosa of the intestine of rats. In the available literature, there are few studies conducted on the same research model. Goda and Takase (1994) [59] showed a reduction in the length of the microvilli (28%) and an increase in their diameter (12%), as well as a reduction in the area of microvilli in enterocytes in animals fed with a high-fat diet. The research of Arija et al., (2000) [32] on different research model also confirmed a number of pathological changes in the intestinal mucosa of chickens fed with a high-fat diet. The authors showed the atrophy and thickening of intestinal villi; the degeneration of enterocytes; as well as the hypertrophy of the lamina propria, goblet cells, intestinal crypts, and the muscular membrane of the jejunum in these animals.

The research carried out in this study showed that, under the influence of heat treatment of garlic, its negative impact on the gastrointestinal wall can be significantly reduced, by significantly reducing degenerative changes in the intestinal mucosa of rats fed with an atherogenic diet, while maintaining a high content of bioactive ingredients (Table 3). Thus, the selection of the appropriate type of heat treatment of garlic can preserve its hypolipidemic properties (Figure 1) and provide effective anti-atherosclerotic effects, while reducing the toxic effects of fresh garlic used for prophylactic and therapeutic purposes over a long period of time.

## 4. Conclusions

The conducted research showed a decrease in the content of total polyphenols, flavonoids, and flavanols, as well as in the antioxidant activity during the prolonged heat treatment of garlic. The highest content of these ingredients and the highest antioxidant potential was characteristic of garlic subjected to short-term heat treatment, such as microwave or blanching, while the lowest was fried, boiled, and additionally microwaved garlic. Short-term heat-treated garlic used in an atherogenic diet, improved the lipid profile of rats’ plasma. Extending the heat treatment time and, in addition, the microwaving of garlic may weaken its action in the body, while still maintaining its hypolipidemic properties which are important in the prevention of cardiovascular diseases.

The greatest influence on the structural changes in the mucosa of the rats’ ileum, manifested by degeneration of the tunica mucosa, shortening of the intestinal villi, damage to the brush border, and thus impairment of the intestinal absorption functions, was that the freeze-dried garlic subjected to short-term heat treatment was used in an atherogenic diet, while blanching was the least beneficial, and longer-lasting thermal processes, such as boiling and frying for 10 min, had a positive effect on the rat intestinal tunica mucosa. Therefore, it can be assumed that compounds negatively affecting the intestinal mucosa, present in fresh or in subjected only to preliminary or short-term (90 s) heat treatment of garlic, are thermolabile and are gradually deactivated under the influence of thermal treatments, thanks to which their toxicity gradually decreases.

The research results obtained in this study provide evidence that the selection of an appropriate method of heat treatment of garlic, also among those used in traditional cooking, may allow the preventive and therapeutic efficacy of garlic to be maintained in cardiovascular diseases, in particular atherosclerosis. At the same time, it can ensure greater safety of long-term use of garlic by minimizing its negative impact on the digestive system.

## Figures and Tables

**Table 1:** Garlic heat treatment processes.

Abbreviation	Garlic Heat Treatment
GF	Freeze-dried fresh garlic
G90s	Freeze-dried 90 s blanched garlic
G10P	Freeze-dried 10 min pan fried without fat garlic
G10	Freeze-dried 10 min boiled in water garlic
GF micro	Freeze-dried fresh and microwaved garlic
G90s micro	Freeze-dried 90 s blanched and microwaved garlic
G10P micro	Freeze-dried 10 min pan fried without fat and microwaved garlic
G10 micro	Freeze-dried 10 min boiled in water and microwaved garlic

**Table 2:** The arrangement of the experimental groups.

Abbreviation	Experimental Group
C	Control group
CH	Control group with 1% of cholesterol
CHGF	Group with 1% of cholesterol and freeze-dried fresh garlic addition
CHG90s	Group with 1% of cholesterol and freeze-dried 90 s. blanched garlic addition
CHG10P	Group with 1% of cholesterol and freeze-dried 10 min pan fried without fat garlic addition
CHG10	Group with 1% of cholesterol and freeze-dried 10 min boiled in water garlic addition
CHGF micro	Group with 1% of cholesterol and freeze-dried fresh and microwaved garlic addition
CHG90s micro	Group with 1% of cholesterol and freeze-dried 90 s blanched and microwaved garlic addition
CHG10P micro	Group with 1% of cholesterol and freeze-dried 10 min pan fried without fat and microwaved garlic addition
CHG10 micro	Group with 1% of cholesterol and freeze-dried 10 min boiled in water and microwaved garlic addition

**Table 3:** The content of selected bioactive compounds and the antioxidant activity (ABTS) of fresh and thermally processed garlic (*Allium sativum* L.).

Sample	Total Polyphenols [mg GAE/g d.m.]	Flavonoids [mg CE/g d.m.]	Flavanols [µg CE/g d.m.]	ABTS [µmol TE/g d.m.]
GF	19.40 ± 1.12 ^c^	3.37 ± 0.31 ^c^	6.71 ± 0.41 ^f^	47.73 ± 1.69 ^c^
G90s	16.11 ± 1.11 ^b^	3.15 ± 0.19 ^c^	5.16 ± 0.38 ^d^	38.77 ± 1.61 ^b^
G10P	15.98 ± 0.98 ^b^	2.94 ± 0.18 ^bc^	4.29 ± 0.31 ^c^	38.92 ± 1.21 ^b^
G10	13.52 ± 0.81 ^a^	1.84 ± 0.12 ^a^	3.99 ± 0.20 ^b^	30.43 ± 1.19 ^a^
GF micro	18.43 ± 0.77 ^c^	3.20 ± 0.25 ^c^	5.88 ± 0.36 ^e^	44.43 ± 1.35 ^c^
G90s micro	15.53 ± 0.89 ^b^	2.37 ± 0.21 ^b^	5.06 ± 0.39 ^d^	35.01 ± 1.51 ^b^
G10P micro	15.11 ± 0.78 ^b^	2.29 ± 0.19 ^b^	3.86 ± 0.39 ^b^	37.76 ± 1.20 ^b^
G10 micro	12.05 ± 0.81 ^a^	1.76 ± 0.12 ^a^	3.06 ± 0.21 ^a^	27.16 ± 1.19 ^a^

Values are means ± SD (*n* = 6). ^a–f^—means in the same columns with different letters are significantly differ (*p* < 0.05); Tukey’s test. d.m.—dry matter; GAE—gallic acid equivalent; CE—catechin equivalent; TE—Trolox equivalent; ABTS—2,2′-Azino-bis-(3-ethyl-benzothiazoline-6-sulfonic acid) diammonium salt).

**Table 4:** Influence of freeze-dried fresh and thermally processed garlic on performance: body weight gain, FER and somatic index of liver (SI-L) in rats fed control- and cholesterol-containing (1% of cholesterol) diet during the 28 day experiment.

Group	Feed Intake [g]	Cholesterol Intake [g]	Garlic Intake [mg]	BWG [g BW]	FER [g/g BW]	SI-L [% BW]
C	590 ± 68 ^ab^	-	-	178.2 ± 27.0 ^bcd^	3.33 ± 0.34 ^a^	3.65 ± 0.37 ^a^
CH	501 ± 36 ^a^	5.0 ± 0.4 ^a^	-	134.9 ± 26.2 ^ab^	3.80 ± 0.61 ^a^	3.70 ± 0.38 ^a^
CHGF	481 ± 42 ^a^	4.8 ± 0.4 ^a^	579 ± 50 ^a^	113.9 ± 27.0 ^a^	4.32 ± 0.54 ^a^	3.26 ± 0.31 ^a^
CHG90s	501 ± 20 ^a^	5.0 ± 0.2 ^a^	602 ± 28 ^a^	127.4 ± 14.6 ^ab^	3.96 ± 0.34 ^a^	3.44 ± 0.37 ^a^
CHG10P	531 ± 23 ^a^	5.3 ± 0.2 ^a^	642 ± 31 ^a^	143.2 ± 18.6 ^abc^	3.74 ± 0.37 ^a^	3.48 ± 0.63 ^a^
CHG10	525 ± 42 ^a^	5.2 ± 0.4 ^a^	635 ± 53 ^a^	136.2 ± 31.0 ^ab^	3.98 ± 0.76 ^a^	3.42 ± 0.66 ^a^
CHGF micro	661 ± 17 ^b^	6.6 ± 0.2 ^b^	746 ± 21 ^b^	198.0 ± 23.9 ^cd^	3.38 ± 0.38 ^a^	3.68 ± 0.35 ^a^
CHG90s micro	665 ± 11 ^b^	6.6 ± 0.1 ^b^	752 ± 14 ^b^	203.7 ± 22.7 ^d^	3.29 ± 0.34 ^a^	3.86 ± 0.16 ^a^
CHG10P micro	666 ± 13 ^b^	6.7 ± 0.1 ^b^	752 ± 16 ^b^	206.7 ± 28.0 ^d^	3.27 ± 0.45 ^a^	3.48 ± 0.46 ^a^
CHG10 micro	663 ± 18 ^b^	6.6 ± 0.2 ^b^	745 ± 20 ^b^	197.8 ± 13.1 ^cd^	3.36 ± 0.14 ^a^	3.55 ± 0.08 ^a^

Values are means ± SD (*n* = 6). ^a–d^—means in the same columns with different letters are significantly differ (*p* < 0.05); Gomes-Howell test: feed intake; Tukey’s test: cholesterol intake, garlic intake, BWG, FER, SI-L. BWG—body weight gain, FER—feed efficiency ratio; SI-L—somatic index of liver.

## Data Availability

Not applicable.
